# Understanding the Neurocomputational Mechanisms of Antidepressant Placebo Effects

**DOI:** 10.20900/jpbs.20210001

**Published:** 2021-02-15

**Authors:** Marta Peciña, Alexandre Y. Dombrovski, Rebecca Price, Helmet T. Karim

**Affiliations:** Department of Psychiatry, University of Pittsburgh, Pittsburgh, PA, 15213, USA

**Keywords:** antidepressant placebo effects, theta burst stimulation, μ-opioid system, reinforcement learning, reward prediction error, ventromedial prefrontal cortex, ventral striatum

## Abstract

Over the last two decades, neuroscientists have used antidepressant placebo probes to examine the biological mechanisms implicated in antidepressant placebo effects. However, findings from these studies have not yet elucidated a model-based theory that would explain the mechanism through which antidepressant expectancies evolve to induce persistent mood changes. Emerging evidence suggests that antidepressant placebo effects may be informed by models of reinforcement learning (RL). Such that an individual’s expectation of improvement is updated with the arrival of new sensory evidence, by incorporating a reward prediction error (RPE), which signals the mismatch between the expected (expected value) and perceived improvement. Consistent with this framework, neuroimaging studies of antidepressant placebo effects have demonstrated placebo-induced μ-opioid activation and increased blood-oxygen-level dependent (BOLD) responses in regions tracking expected values (e.g., ventromedial prefrontal cortex (vmPFC)) and RPEs (e.g., ventral striatum (VS)). In this study, we will demonstrate the causal contribution of reward learning signals (expected values and RPEs) to antidepressant placebo effects by experimentally manipulating expected values using transcranial magnetic stimulation (TMS) targeting the vmPFC and μ-opioid striatal RPE signal using pharmacological approaches. We hypothesized that antidepressant placebo expectancies are represented in the vmPFC (expected value) and updated by means of μ-opioid-modulated striatal learning signal. In a 3 × 3 factorial double-blind design, we will randomize 120 antidepressant-free individuals with depressive symptoms to one of three between-subject opioid conditions: the μ-opioid agonist buprenorphine, the μ-opioid antagonist naltrexone, or an inert pill. Within each arm, individuals will be assigned to receive three within-subject counterbalanced forms of TMS targeting the vmPFC—intermittent Theta Burst Stimulation (TBS) expected to potentiate the vmPFC, continuous TBS expected to de-potentiate the vmPFC, or sham TBS. These experimental manipulations will be used to modulate trial-by-trial reward learning signals and related brain activity during the Antidepressant Placebo functional MRI (fMRI) Task to address the following aims: (1) investigate the relationship between reward learning signals within the vmPFC-VS circuit and antidepressant placebo effects; (2) examine the causal contribution of vmPFC expected value computations to antidepressant placebo effects; and (3) investigate the causal contribution of μ-opioid-modulated striatal RPEs to antidepressant placebo effects. The proposed study will be the first to investigate the causal contribution of μ-opioid-modulated vmPFC-VS learning signals to antidepressant placebo responses, paving the way for developing novel treatments modulating learning processes and objective means of quantifying and potentially reducing placebo effects during drug development.

**Trial Registration:**

ClinicalTrials.gov Identifier: NCT04276259.

## SIGNIFICANCE

Major Depressive Disorder (MDD) affects ~16 million adults in the U.S. and is the leading cause of disability [1]. Modest responses to antidepressant treatments (~50%) are also characterized by high placebo response rates (~31%) [2], which contribute to the failure of antidepressant clinical trials and discourage new investments for novel antidepressant targets [3]. Therefore, understanding the mechanisms underlying placebo responses is essential to explain antidepressant treatment response variability and to identify novel therapeutic targets for depression.

For modern medicine, placebos provide a window into internal brain processes that influence health. Over the last two decades, neuroscientists have used antidepressant placebo probes to examine the biological mechanisms through which antidepressant expectancies [4] motivate sustained mood responses [5-8]. Recent neuroimaging studies of antidepressant placebo effects have further demonstrated placebo-induced increased μ-opioid signaling [5] and BOLD responses in areas involved in cognitive control [6] (e.g., dorsolateral prefrontal cortex), the representation of expected values [7] (e.g., vmPFC), reward [9] (e.g., VS) and emotional processing [5]. These studies have demonstrated the biological mechanisms underlying antidepressant placebo effects, they have yet to describe a mechanism through which antidepressant expectancies evolve to induce persistent mood changes, like those observed in randomized clinical trials (RCT). More specifically, no study has interrogated antidepressant placebo effects from a theory-driven perspective with a rigorous computational approach that would parametrize individual differences in placebo responses. The estimation of such computational parameters which cannot be accessed with descriptive approaches alone provides new opportunities to disambiguate placebo responses.

## RL

Classical theories of the placebo effect, using analgesia experiments, have shown that placebo responses are explained predominantly by expectancy and conditioning mechanisms [4]. While oftentimes both mechanisms work synergistically, the former understands placebo effects as a product of expectations (e.g., “verbal instructions”), whereas the latter understands them as conditioned responses through the pairing of a neutral stimulus (e.g., the placebo pill) with an unconditioned stimulus (e.g., the active drug). More recently, RL theories [10] have provided a new explanatory framework, essentially integrating the expectancy and conditioning theories, where learning does not only depend on simple contiguity between the conditioned and unconditioned stimuli, but on RPE, which signals mismatch between what it is expected (*expected value*) and what it is experienced. In standard RL, expectations not reinforced by experience are extinguished. However, emerging evidence from placebo analgesia experiments suggests that placebo analgesia might be explained *self-reinforcing expectancies* mechanisms, such as *confirmation biases*, where expectancies are selectively reinforced by predictive cues (e.g., the placebo) only when new experience confirms prior expectations [11]. Alternatively, others have suggested that persistent expectancies result from *impaired extinction learning* caused by prefrontal downregulation of reward signals [12]. Furthermore, consistent with RL theories of placebo effects, these studies have demonstrated placebo-induced activation in several cortical areas implicated in the representation of expected values, such as the vmPFC, and subcortical areas implicated in reward processing, such as the VS [13]. While a computational framework of placebo analgesia is yet to be fully developed, these new insights provide promising evidence that placebo analgesia conforms to models of RL, a theoretical framework that will likely apply to other clinical conditions, such as depression. Yet, the role of RL in antidepressant placebo effects has never been tested.

## THE CENTRAL HYPOTHESIS

In line with a RL theory of antidepressant placebo effects, the central hypothesis of this application is that antidepressant placebo expectancies are tracked by the vmPFC and updated by means of μ-opioid-modulated striatal RPE signal.

### The Ventromedial Prefrontal Cortex

The vmPFC (defined here as the ventral medial cortex and the adjacent medial orbitofrontal cortex) has been robustly involved in the formation of placebo analgesia [14-16] and antidepressant placebo effects [7,8,17]. Beyond placebo effects, the vmPFC cortex has been implicated in a variety of cognitive, social, and affective functions, as well as in the neurobiology of depression and mechanisms of treatment response [18], including the prediction of treatment response across a wide range of treatment modalities [19]. In particular, the vmPFC has been involved in value-based decision making, including RL [20], the regulation of negative emotion and the processing of self-relevant information [18]. Furthermore, it has been argued that the vmPFC is not necessary for affective responses per se, but is critical when affective responses are shaped by conceptual information (“meaning”) about specific outcomes [21]. Therefore, the vmPFC appears as a modulatory target of antidepressant placebo effects.

### The μ-Opioid System and RPE

Neuropharmacological studies using μ-opioid antagonists [22-26] and measures of in vivo μ-opioid receptor availability [27,28] have conclusively implicated μ-opioid neurotransmission in placebo analgesia. μ-Opioid receptors, the primary site of action of endogenous opioid peptides [29], are widely distributed in the brain and attain their highest levels in the prefrontal cortex, VS, thalamus and the amygdala [30].

In the first study that examined the molecular correlates of fast-acting antidepressant effects, we used positron emission tomography and the μ-opioid receptor radiotracer [^11^C]carfentanil in 35 patients with MDD [5]. This study demonstrated that the improvement of depressive symptoms in response to i.v. placebo with expectations of fast-acting antidepressant effects was positively correlated with the release of endogenous opioids in the VS and vmPFC, among other regions. Placebo-induced mood improvement and opioid release in these regions predicted up to 43% of the variance in the clinical response to 10 weeks of open-label antidepressant treatment. A subset of this sample (*n* = 26) also completed the same study with the D_2/3_ receptor radiotracer [^11^C] raclopride. In this case, the administration of the placebo was also associated with increased striatal dopamine release, however, *striatal dopamine release was not associated with placebo- or antidepressant-induced mood improvement* [9]. Consistently and as suggested by prominent reward theories [31], while both neurotransmitter systems may be released in response to the administration of placebos, the mesolimbic dopamine system may be involved in the placebo “wanting” or the motivation to obtain a placebo reward, while opioids may be involved in the physiological response to a hedonic stimuli or placebo “liking”.

The common assumption about the role of opioids in placebo effects is that they are released in response to expectancies and act as endogenous analgesic. An alternative account from studies of conditioned analgesia [32,33] posits that in addition to the direct effect of opioids in sensory perception (e.g. pain, mood), opioid modulates learning by reducing the discrepancy between the expected values and the reward [34] or by modulating the sensitivity to reward. However, these hypotheses have yet to be tested.

**Figure 1.**
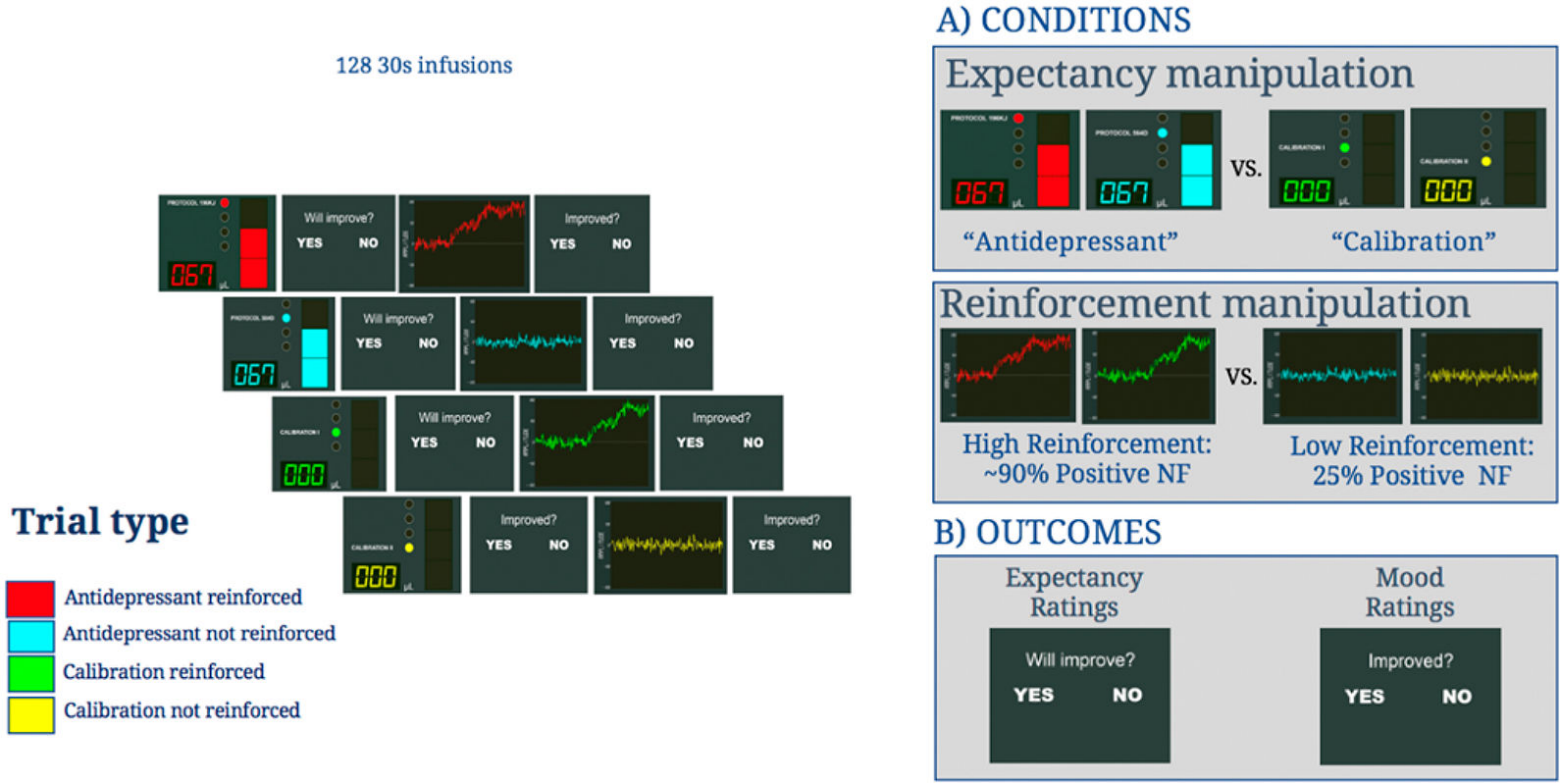
The Antidepressant Placebo fMRI Task.

Building on the evidence describe above, in this application we will use a novel Antidepressant Placebo fMRI Task (see Approach & Figure 1) developed by our group to examine how placebo-induced expectations of mood improvement and their reinforcement by sham neurofeedback, to build a computational model of antidepressant placebo effects. In addition, we will use TMS and pharmacological opioid modulation to manipulate reward learning signals (e.g., expected values and RPEs) resulting from the computational models of antidepressant placebo effects. Specifically, in a 3 × 3 factorial double-blind design, we will randomize 120 antidepressant-free individuals with depressive symptoms (18–55 years) to one of three between-subject opioid conditions: the μ-opioid agonist buprenorphine (n = 40), the μ-opioid antagonist naltrexone (*n* = 40), or an inert pill (*n* = 40). Within each arm, individuals will be assigned to receive three within-subject counterbalanced forms of TMS targeting the vmPFC—intermittent Theta Burst Stimulation (iTBS) expected to potentiate the vmPFC, continuous TBS (cTBS) expected to de-potentiate the vmPFC, or sham TBS (sTBS). These experimental manipulations will be used to modulate trial-by-trial reward learning signals and related brain activity during the Antidepressant Placebo Task to address the following aims:

**AIM 1:** Investigate the relationship between reward learning signals computations within the vmPFC-VS circuit and antidepressant placebo effects. During the Antidepressant Placebo fMRI task, H1a: antidepressant placebos will enhance the representation of reward learning signals (expected values and RPEs) in the vmPFC-VS circuit; H1b: Increased neural learning signals will enhance mood improvement.

**AIM 2:** Examine the causal contribution of vmPFC expected value computations to antidepressant placebo effects. Compared to sTBS, H2a: vmPFC iTBS (potentiation) will increase expected value representation in the vmPFC-VS circuit, enhancing mood improvement, whereas H2b: cTBS (de-potentiation), will induce the opposite effects.

**AIM 3:** Investigate the causal contribution of μ-opioid-modulated RPEs to antidepressant placebo effects. Compared to the inert pill condition, H3a: the partial μ-opioid agonist buprenorphine will be associated with increased striatal RPEs, enhancing mood improvement, whereas H3b: the μ-opioid antagonist naltrexone will induce the opposite effects.

The proposed study will be the first to investigate the causal contribution of μ-opioid-modulated reward learning signals within the vmPFC-VS circuit to antidepressant placebo responses. Insights from this study could have a transformative impact on our understanding of antidepressant treatment effects and pave the way for developing novel treatments modulating learning processes (vmPFC iTBS/ Buprenorphine) and objective means of quantifying or potentially reducing placebo effects during drug development.

## IMPACT

Placebos are powerful tools that modern medicine has often overlooked. Research over the last four decades has demonstrated that placebo effects induce physiological and neural changes that lead to symptom improvement (e.g., pain, mood, itch) [35,36]. Definitive studies of the brain pathways involved in placebo responses are therefore critical for understanding placebo effects; identifying biomarkers of treatment response; elucidating new targets for drug development; and improving assay sensitivity in antidepressant clinical trials. This proposal addresses these questions by combining a computational psychiatry framework and novel experimental manipulations to delineate the computational, neural, and molecular mechanisms that causally contribute to placebo-induced mood improvement. This learning framework represents a shift in paradigm, where expectancies associated with treatment cues are understood as conditioned stimuli with the ability to induce conditioned responses that modify behavior. Under these theories, placebos and treatment cues broadly (e.g., injections, devices), are no longer inert treatments, but predictive cues with the potential to be learned and modulated to promote treatment response.

The delineation of the computational framework and associated neural circuits and neurotransmitters systems that explains antidepressant placebo effects opens new translational opportunities to promote treatment response. In this application, we propose harnessing placebo responses, using TBS potentiation or opioid stimulation approaches. The stimulation of placebo-related networks may result in new targets for mood modulation. This approach may be especially important in conditions such as Treatment-Resistant Depression, where failure to multiple lines of treatment could be explained by dysfunctions in reward learning processing, explaining why μ-opioid modulation has proven to be a successful treatment in resistant depression [37,38]. Furthermore, from the perspective of drug development, inhibiting placebo responses using TBS depotentiation or μ-opioid blockade, could help separate drug-specific and “non-specific” treatment effects, and result in substantial savings by reducing the samples sizes necessary to achieve significant differences between active and inactive treatments.

## INNOVATION

To our knowledge, the proposed study is the first attempt to examine the causal contribution of RL theories to antidepressant placebo effects. While recent evidence suggests that RL theories play a significant role in placebo analgesia [11,12], similar theories have never been tested in the field of antidepressant placebo effects, and promise to transform how we understand, enhance, inhibit and control for antidepressant placebo effects. Furthermore, this transdiagnostic RL framework may apply to other clinical conditions where placebo effects are also prevalent, such us anxiety disorders, schizophrenia and substance use disorders [2,39,40]. To attain this scientific aim, we propose a series of methodological innovations that will quickly accelerate and transform our current understanding of how antidepressant expectancies are learned to promote mood changes. First, we will use a trial-by-trial manipulation of antidepressant placebo effects (Antidepressant Placebo fMRI Task). This trial-by-trial manipulation was essential to decoding the neural representation of placebo effects, by aiding the development of computational models which, in turn, estimate the trial-by-trial fluctuation of reward learning signals. We will implement these analyses using state-of-the-art hierarchical Bayesian approaches [41]. Second, we will modulate reward learning signals using vmPFC TBS. Previous studies have demonstrated placebo analgesia blockade using low-frequency dorsolateral prefrontal cortex TMS [42], without regard for its theoretical framework. Here, we will potentiate and de-potentiate a target relevant to the RL framework under investigation, mechanistically demonstrating the implication of the vmPFC-VS circuit in the context of RL theories of antidepressant placebo effects. This approach has been successfully implemented by co-I Price targeting the orbitofrontal cortex in patients with obsessive compulsive disorder. Third, while μ-opioid neurotransmission has been linked to placebo effects using in vivo molecular imaging [5,28,35] and opioid blockade [26], the potential for enhancing placebo effects using μ-opioid partial agonist has never been tested. Furthermore, the role of the μ-opioid within RL theories of antidepressant placebo effects still needs to be established. Overall, we will combine RL model-based fMRI, pharmacological opioid manipulation and vmPFC neuromodulation to test, for the first time, the causal contribution of RL to antidepressant placebo effects.

## APPROACH

### Study Design Overview

**Figure 2.**
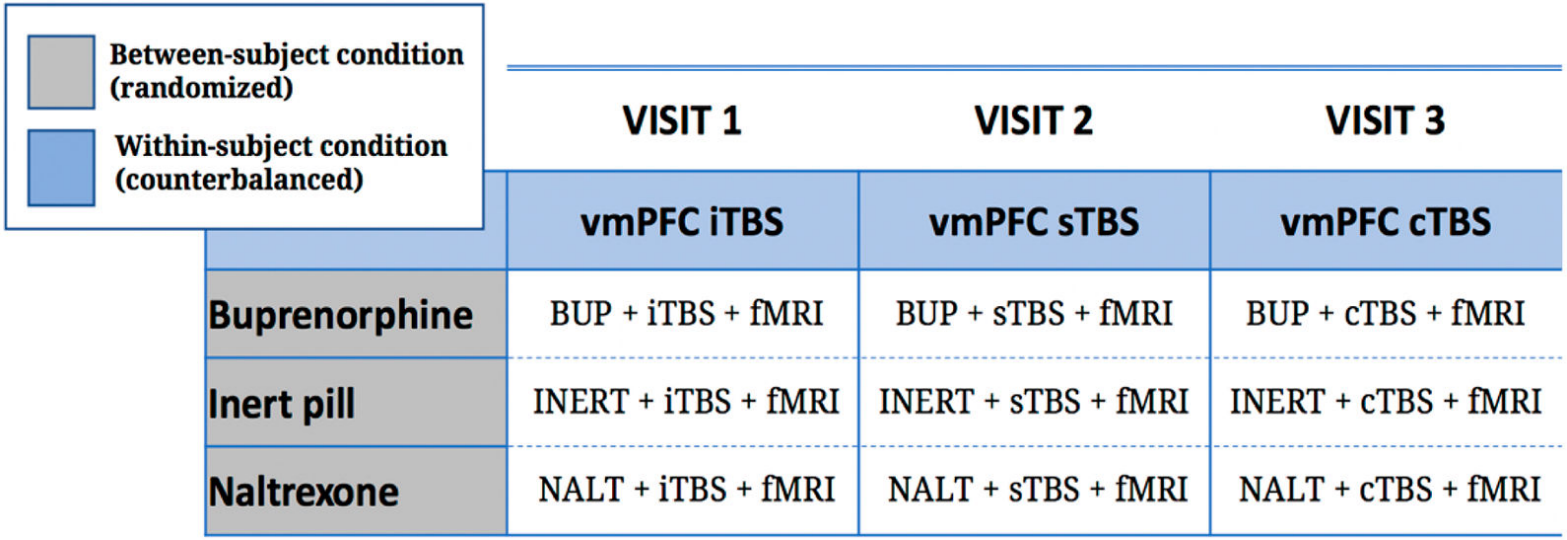
3 × 3 Factorial Study Design of the μ-opioid modulation intervention and the TMS intervention prior to the Antidepressant Placebo fMRI Task (Figure 1).

In a 3 × 3 factorial double-blind trial, we will randomize 120 antidepressant-free individuals with depressive symptoms (18–55 years) to one of three between-subject opioid conditions: the μ-opioid agonist buprenorphine (*n* = 40), the μ-opioid antagonist naltrexone (*n* = 40), or an inert pill (*n* = 40). Within each arm, individuals will be assigned to receive three within-subject counterbalanced sessions of TBS targeting the vmPFC—iTBS expected to potentiate the vmPFC, cTBS expected to de-potentiate the vmPFC, and sTBS (Figure 2). These experimental manipulations will be used to modulate reward learning signals and associated brain responses during the Antidepressant Placebo fMRI Task (Figure 2).

### Participants

We will recruit 120 antidepressant-free individuals with depressive symptoms (ages 18–55; approx. 60% female) through referrals from clinics in the area, the Student Health Service, and the Research Participant Registry, funded by the National Institutes of Health and maintained by the Clinical and Translational Science Institute of the University of Pittsburgh. To ensure excellent follow-up retention, we will (1) collect contact information for one individual who knows the participant, (2) maintain regular contact with participants, and (3) use electronic search services to update contact information. Following an initial screening, participants will be invited to an in-person visit to sign the consent form, confirm eligibility after evaluating the inclusion/exclusion criteria and suicidal risk, collect clinical data and conduct a drug and pregnancy test.

#### Core clinical assessments

We propose sampling across the full dimension of the anhedonic depression symptomatology. We will use the Mood and Anxiety Symptom Questionnaire (MASQ) [43]. The MASQ is a 62-item self-report questionnaire that assesses depressive, anxious, and mixed symptomatology using three different facets: (1) General Distress; (2) Anxious Arousal), and (3) Anhedonic Depression. Higher scores reflect greater levels of symptomatology. A cut-off of 23 in the Anhedonic Depression facet is used to diagnose caseness for Mood Disorders [44]. We propose to recruit 2/3 of the sample above this cut-off. In addition, we will recruit 1/3 of the sample below this cut-off to ensure the full dimension of anhedonic depression symptomatology.

In addition, participants will complete two depression severity scales: the clinician administered Montgomery-Åsberg Rating Scale [45], and the self-reported Quick Inventory of Depressive Symptomatology [46]. Because of our interest in reward processing and MDD, we will investigate two facets of reward-guided behavior [47] commonly affected in MDD: motivation, using the Apathy Evaluation Scale [48], and hedonic state, using the Snaith-Hamilton Pleasure Scale [49]. In addition, we will collect information about personality traits using the Revised NEO Personality Inventory [50], history of trauma using the Childhood Trauma Questionnaire [51] and anxiety comorbidity for exploratory analysis.

#### Randomization and blinding procedures

We will use an in-house MatLab software to randomly assign participants into one of three between-subject opioid conditions: the μ-opioid agonist buprenorphine, the μ-opioid antagonist naltrexone, or the inert pill. We will use dynamic/adaptive randomization to account for age, sex, and MASQ scores differences at baseline. We will similarly counterbalance the order in which participants receive iTBS, cTBS, and sTBS using a similar procedure. We will ensure that the rate at which participants are assigned to any group or order will not differ significantly between the opioid and TBS conditions. Fifty percent of subjects will be assigned to sham TBS simulating the iTBS stimulus pattern and the other 50% will be assigned to sham TBS simulating the cTBS stimulus pattern. Participants, PI and staff member will be blinded to the study procedures. Only one staff member will be unblinded to all study procedures. This person will also deliver the TBS.

#### Study timeline

After randomization, eligible participants will complete three opioid/TBS/fMRI visits (each ~150 min) on three different days (~5–10 days apart). To avoid the delayed onset of antidepressant treatment, study participation will be completed in ~4 weeks, and participants will be instructed to arrange post-participation follow-up care with a psychiatrist at baseline. At each visit, participants will complete: a pregnancy test; the TBS; the administration of the opioid/placebo; the intravenous (I.V.) placement; the pre-scan expectancy questionnaires; the Antidepressant Placebo fMRI Task; and the post-scan effectiveness and credibility questionnaires. To ensure safety, participants will remain in observation for ~30 min after scanner completion.

#### Study sample considerations

We considered recruiting a clinical control sample (e.g., obsessive compulsive disorder) to ensure that our findings were disease-specific but decided that this aim would be most appropriate for a follow-up study and we limited recruitment to antidepressant-free individuals with depressive symptoms. We excluded participants below age 18 and above age 55 to avoid age-related confounders (e.g., neurodevelopment, neurodegenerative diseases and/or vascular pathology). We also considered excluding young adults (18–25) to prevent confounders involved in brain maturation but opted for a wide age range (18–55), to facilitate recruitment and maintain age-range consistency with current studies. Furthermore, our pilot studies show no behavioral effects of age on placebo-induced expectancy and mood responses. Finally, we considered recruiting medicated individuals with depressive symptoms but chose to study antidepressant-free individuals with depressive symptoms to avoid potential confounding effects of psychotropic medication.

#### Alternative study designs and eligibility criteria

We considered a fully within-subject 6-condition design (iTBS vs cTBS vs sTBS vs buprenorphine vs naltrexone vs inert pill), but opted for a 3 × 3 factorial design to avoid undermining the credibility of the placebo intervention by repeated administrations of the Antidepressant Placebo Task (×6) and to reduce attrition to the study. Furthermore, the present study design allows us to test for TBS*opioid interaction effects in exploratory analyses. We also considered a 3 × 3 mixed factorial design with TBS sessions as the between-subject conditions but opted for TBS as the within-subject condition to improve TBS tolerability. Finally, we considered an interleaved TBS/fMRI, but chose to measure post-TBS effects, as proposed TBS protocols are readily clinically translatable and cost-effective, taking full advantage of our TMS equipment.

### Study Interventions

#### Opioid modulation

Within each condition, all participants will receive one intramuscular (I.M.) arm injection and one oral tablet. In the buprenorphine condition, participants will receive one I.M. injection of 0.3 mg/1 mL buprenorphine hydrochloride (Buprenex®; Richmond, VA: Reckitt Benckiser Pharmaceuticals Inc.; 2006) (onset of action: ≥15 min; peak effect: ~1 h; duration: ~6 h) and an oral placebo tablet. Buprenorphine is a μ-opioid partial agonist and kappa-opioid antagonist that is used to treat moderate to severe pain and opioid dependence. Notably, the dose proposed in this study is less than one-twentieth of the one used in opioid replacement therapy. Studies using a similar dose (0.2 mg sublingual) have shown to improve memory for social reward [52], reduce fear recognition [53], reduce attention bias to emotive faces and responses to emotional images [54] without producing appreciable subjective effects or nausea. In the naltrexone condition, participants will receive one oral tablet of 50mg naltrexone hydrochloride (ReVia®; Toronto, ON: Teva Canada Limited; 2015) (onset of action: ≥15 min; peak effect: ~1 h; duration: ~24 h) and a saline I.M. arm injection. Naltrexone is thought to strongly block μ-opioid receptors [55]. A dose of 50 mg is considered an effective dose for the treatment of drug dependence [56] and the dose most commonly used to examine drug effects on reward processing in healthy adults [57]. We have successfully administered one dose of naltrexone 50 mg with significant drug effects on reinforcement-induced mood changes, RPEs, and placebo-induced neural responses, and reasonable tolerability, with the most common side effect being nausea and/or fatigue (60% of patients on naltrexone compared to 36% of patients on placebo). In the inert pill condition, participants will receive one I.M. arm injection of saline (1 mL) and an oral placebo tablet. Alternative doses: We considered using sublingual buprenorphine (0.2 mg), for easier administration. However, sublingual buprenorphine peaks 90 to 360 min after ingestion [58], which is significantly different from naltrexone’s peak at ~1 h. Furthermore, compared to sublingual buprenorphine, which needs to be imported, I.M. buprenorphine hydrochloride is readily available at the University of Pittsburgh’s Investigational Drug Services.

#### vmPFC theta burst stimulation

TMS is an FDA approved treatment for depression and other psychiatric conditions and is extensively used in research to induced performance enhancement [59]. More recently, TBS is being used to briefly and effectively manipulate brain function in opposing directions, non-invasively [60,61]. TBS creates a 50–60 min window of potentiation (with intermittent 2-second pulses or “bursts” of stimulation; iTBS) or depotentiation (with continuous 2s pulses; cTBS) using a potent, very brief (40–120 s total) approach. Research has shown that vmPFC cTBS attenuates neural reactivity to drug and alcohol cues in frontostriatal circuits [60], yet to our knowledge, there is no evidence of the effects of TBS on value representation. The efficacy of TBS to successfully modulate medioprefrontal regions has also been well-established through fMRI assessments, using a similar approach [60]. Here, we propose to use iTBS and cTBS to potentiate and depotentiation value representation of the expected improvement in the vmPFC.

##### Choosing Target:

**Figure 3.**
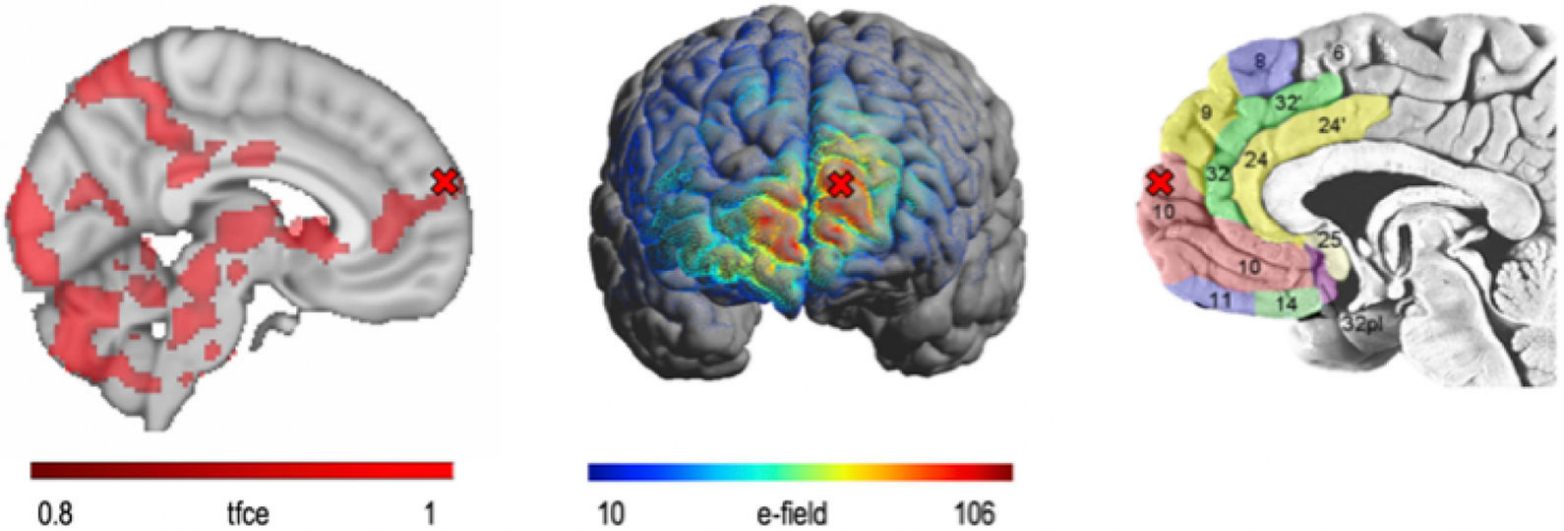
vmPFC Target. We will target an anterior/medial junction to modulate right hemisphere vmPFC (BA 10), which can be reached with a standard TMS coil (**right**). This coordinate was found to be the most cortical portion of the vmPFC activated during the Antidepressant Placebo fMRI Task (**left**). The electrical field maps confirm good coverage of the target region (**middle**).

Within each group, participants will receive three counterbalanced forms of TMS targeting the vmPFC (within-subject TMS condition)—iTBS, expected to potentiate the vmPFC, cTBS, expected to de-potentiate the vmPFC, or sTBS. Target location: The location of M1 will be identified using a trial-and-error process and then then the motor threshold is established, which is the minimum stimulation required to move the thumb/index finger 5 consecutive times. The right vmPFC (BA 10, Figure 3) will be identified using the 10–20 electroencephalogram coordinate system, which has been used to reliably locate regions like the dorsolateral prefrontal cortex [62]. The TMS coil will be positioned over the MNI coordinate 6, 60, 28 and will be oriented to optimize the electrical field over the cortical region. We acknowledge numerous promising treatment targets for antidepressant placebo effects outside the vmPFC (e.g., dorsolateral prefrontal cortex, as previously described in placebo analgesia studies [42]), but opted for the vmPFC on the basis of our interest in value representation, which is strongly linked to the vmPFC, and the activation of this region on our Antidepressant Placebo fMRI task. Alternatively, we could have opted to stimulate the ventrolateral prefrontal cortex, also activated during our task, however, this region was unilateral and significantly closer to the periorbital area, and therefore its stimulation is potentially less well tolerated. Safety and tolerability of iTBS and cTBS targeting vmPFC have been established in healthy volunteers [63] and in clinical populations [61].

##### Choosing Frequency:

Dosing was informed by meta-analyses of iTBS and cTBS protocols delivered over the motor cortex [64], suggesting reliable increases (iTBS) and decreases (cTBS) in motor evoked potentials for 50–60 min, with large effect sizes peaking 10–15 min post-TBS. Previous research has shown that vmPFC cTBS attenuates neural reactivity to drug cues in fronto-striatal regions [65]. Our calculations confirm comparable electrical field exposure compared to the motor cortex, and electrical field estimates confirm good coverage of the target region. After determining resting motor threshold (RMT), the TMS coil will be positioned over the idiographic navigational system-identified vmPFC target. Participants will receive two blocks of each TBS form. During the first block, stimulation intensity will be gradually escalated in 5% increments (from 30% to 110% RMT) in order to enhance tolerability. In all conditions, we will apply 600 pulses/block of theta burst (bursts of three stimuli at 50 Hz repeated at 5 Hz frequency) at 110% RMT (as in the prior vmPFC cTBS study [60]). Each block of iTBS will consist of 20 trains, each lasting 2 s with intertrain intervals of 8 s, for a total of 192 s. Each block of cTBS will consist of one continuous train of 40s. A rigorous active sTBS, as previously described [65], will make use of two surface electrodes placed on the scalp (present for both real and sham TBS but activated only during sham) and the participant’s assigned sham TBS protocol will be run while the TMS coil is flipped 180 degrees, generating an identical pattern, sound, and pressure. Sham TBS electrodes will simulate either the stimulation patterns of cTBS or iTBS, and participants will be assigned to either sham condition on a 1:1 basis. We do not anticipate any differences between the sham iTBS or sham cTBS.

#### The antidepressant placebo task

Immediately after the opioid + TBS sessions, participants will undergo the Antidepressant Placebo fMRI Task (Figure 1). Before the scanning session, a certified nurse will place an fMRI compatible I.V. line in the participant’s arm prior to the administration of the “fast-acting” or “conventional” I.V. antidepressant. The I.V. arm-side will be the same for all visits within-subjects but counterbalanced between-subjects. Once in the scanner, an MRI compatible pump, controlled from the scanning room by pushing the “go” trigger, will deliver the saline to the participant during the scanning session. The infusion is manually started at a given flow rate and volume, at the beginning of each run. The task programmed using PsychToolbox-3 software [66] is then presented via a display placed behind the gantry.

The Antidepressant Placebo fMRI Task features two putative components of the placebo effect: the expectancy and reinforcement condition, each followed by an expectancy and mood rating cue, respectively. The expectancy condition involves two “antidepressant” infusion cues described as a “fast-acting” and a “conventional antidepressant” and two no-infusion cues described as periods of equipment calibration. During the “antidepressant” infusion cue (4 s), a bar is filled at four 1 second-periods representing 0%, 33%, 66% and 100% of the dose administered. During the calibration no-infusion cue (4 s) the bar remains empty. In the high-reinforcement condition sham neurofeedback is positive on 88% of the trials and remains at baseline on 12%. In the low-reinforcement condition, sham neurofeedback is positive on 25% of the trials and remains at baseline on 75%. The overall number of trials is 128 (32 trials per run, 8 trials per condition: (1) “antidepressant” reinforced; (2) “antidepressant” not reinforced; (3) calibration reinforced; and (4) calibration not reinforced). The number of positive neurofeedback trials per run in conditions 1 & 3 is 7, compared to 1 baseline neurofeedback trail. The number of positive neurofeedback trials per run in conditions 2 & 4 is 2, compared to 6 baseline neurofeedback trials. Participants rate their expected and actual change in mood (YES/NO) in response to each infusion/neurofeedback signal respectively by using a keypad and their index fingers. To avoid learning effects, participants complete different versions of the task at each visit, where each version will be coded with four different color-types, but identical task structure otherwise.

Authorized deception and instructions to participants: Participants will be fully informed about both the opioid and the TMS modulation, including their pharmacological properties, their general clinical use, and their possible side effects. However, participant’s will not be informed about the purpose of the study—the investigation of antidepressant placebo effects. Instead, during the consent process, participants will be informed that certain aspects of the study will be intentionally mis-described and would be revealed to them at the end of their participation in the study. This authorized deception procedure is commonly used in placebo research [67] and has been successfully used in our previous studies. Specifically, participants will be told that: “We are investigating the effects of a fast-acting antidepressant compared to a “conventional” antidepressant on neural activity. Both drugs will be administered I.V. during multiple consecutive injections while we record your brain activity”. In addition, during their in-person screening visit, participants will watch a fragment of the Antidepressant Placebo fMRI Task while instructed: “A drug-infusion cue will alert you that a new drug infusion is about to start. Each drug infusion will be immediately followed by the displayed of your brain responses. Higher brain signal tracing reflects the effectiveness of the drug infusion and may result in mood improvement, whereas the baseline neurofeedback signal is unlikely to cause mood improvement. While you will receive both, the fast-acting and the conventional antidepressant, at each drug infusion, you will not be informed of the drug type, however you should expect more positive brain responses in response to the fast-acting antidepressant and be able to differentiate between the two. In addition, there will be periods of equipment “calibration”, in which no drug will be administered, but we will continue to record your brain activity”. No drug is ever administered, only saline, and the brain signals displayed are simulated.

Assessment of baseline expectancies (pre-scan expectancy questionnaire): Immediately after describing the placebo intervention but before the fMRI experiment, the investigators’ will evaluate the patient’s baseline experiences with the following questions: How do you think the drug infusions will change your mood? How do you think the calibration periods will change your mood? How do you think the neurofeedback will change your mood?

Assessment of the credibility of the experiment: After the experiment, the investigators will assess the credibility of the placebo manipulation by asking the following questions: From 0 to 100% how often: did the neurofeedback signal reflect your brain activity? Did you receive the fast-acting antidepressant treatment during the infusion periods? and did you receive saline during the calibration periods? Participants who respond 0 to questions 1 and 2 and responded 100 to question 3 will be excluded from the experiment. In our feasibility (*n* = 24) and pilot study (*n* = 35), no participant has been excluded for lack of credibility in the experiment.

fMRI data acquisition, preprocessing and analysis: fMRI data will be collected in a Siemens 3T MAGNETOM Prisma Fit, with a 64-channel coil, using simultaneous multi-slice eco planar imaging acquisition (repetition time = 1000 ms, echo time=30 ms, multiband factor = 5, 2.3 mm^3^ voxels). Data will be preprocessed using fMRIPrep [68], which implements registration methods (incl. ANTS SyN) that maximize inter-subject spatial similarity. We will apply susceptibility correction using FSL TOPUP [69] and mitigate the negative effects of physiological artifacts using RETROICOR [70] and the PhysIO toolbox [71]. Motion artifacts will be handled using ICA-AROMA [72].

### Statistical Analysis

#### AIM 1: To establish a relationship between reward learning signals within the vmPFC-VS circuit and antidepressant placebo effects

The overarching goal of Aim 1 is to establish the neurocomputational mechanisms of antidepressant placebo effects within RL framework. To do so, we will use RL to model trial-by-trial expectancy ratings during Antidepressant Placebo fMRI Task and map the estimated learning signals to the neural responses during the task. Hypothesis and expected outcomes: We hypothesized that during the Antidepressant Placebo fMRI task, H1a: antidepressant placebos will increase the representation of reward learning signals (expected values and RPEs) in the vmPFC-VS circuit (*N* = 120). H1b: Increased neural learning signals will enhance mood improvement (*N* = 120).

##### Statistical Analysis:

###### RL Model:

To obtain expected values and RPEs, we will model learning within the RL theory. RL models track how participants adapt their behavior to maximize rewards by incorporating objective factors, such as prior beliefs and experience. All models will be fit to participant’s behavior using hierarchical Bayesian estimation using Markov chain Monte Carlo sampling implemented in Stan [41]. Learned expected values for each of the four trial conditions of the Antidepressant Placebo Task will be updated every time the “antidepressant” or “calibration” cue is presented and an outcome (positive or baseline neurofeedback) is observed, based on the following equation: *Q*_*t*+1_(*s*) = *Q*_*t*_(*s*)+α*δ*_*t*_, where *Q*_*t*_(*s*) is the learned expected value of improvement *s* at trial *t*, α is a learning rate, and *δ* is the difference between the actual and expected outcome (RPE): *δ*_*t*_ = *r*_*t*_ − *Q*_*t*_(*s*), where, *r*_*t*_ is the actual reward outcome (positive vs baseline neurofeedback). The sigmoid choice rule will include two free parameters: stochasticity and choice bias. Alternative model parametrizations will be tested using Bayesian model comparison [73] with a correction for the omnibus Bayesian error rate [74].

###### Expected values and RPEs computations:

Estimated learning signals (expected values and RPEs) generated from the RL model will be mapped to neural activity. The level 1 model will include four event first-level regressors: infusion event, expectancy rating event, neurofeedback event and mood rating event. We will also include regressors for learned expected value and RPEs aligned with the neurofeedback event as well as their interactions with the expectancy condition (“antidepressant” vs “calibration”). In a model-based voxel-wise general linear model analyses we will assess the main effects of expected value and RPE signals, and their interaction with the expectancy condition in one-sample t-tests using FEAT and randomize [75]. We will employ threshold-free cluster enhancement for type I error control for optimal sensitivity [76]. Mean average regression coefficients for BOLD responses for each of the two regions within the vmPFC-VS circuit will be extracted for statistical analysis. If exploratory voxel-wise analyses are not statistically significant within the vmPFC-VS circuit, we will use a region-of-interest (ROI) approach, focusing on the vmPFC and VS. ROIs coordinates will be obtained from relevant meta-analyses [77,78].

###### vmPFC-VS pathway prediction of mood:

To assess whether neural learning signals within the vmPFC-VS interact with the task conditions to enhance mood ratings (H1b), we will conduct a logistic mixed-effects regression analysis using the lme4 package [79] in *R*. Predictors of mood ratings will include the expectancy condition (“antidepressant” infusion vs. calibration no-infusion cue), reinforcement condition (high vs low reinforcement), neural reward learning signals within the vmPFC-VS circuit and their interactions. Subject and run (clustering within-subject) intercepts will be taken to be random in all models. Significant predictors will be tested using the likelihood ratio test (LRT; car:: Anova). To diagnose multicollinearity among predictors we will calculate variance inflation factors (VIFs) and ensure that all regressors meet a rigorous criterion of VIF < 3.

##### Alternative outcomes:

If the expectancy condition does not enhance reward learning signals in the vmPFC-VS circuit, we will investigate alternative ROIs within the expected value and RPE map (e.g., vlPFC, lateral orbitofrontal cortex, anterior insula). If greater neural learning signals do not enhance, but decrease, mood improvement during the Antidepressant Placebo fMRI Task, we will investigate the possibility that placebo effects involve diminished extinction learning (e.g., by testing RL models embodying this hypothesis against behavioral/neural data).

Power analysis (pw [80] and WebPower [81] packages in *R*). We will investigate Aim 1 in the entire sample (*N* = 120) during the sTBS condition only, while separately controlling for the effects of the opioid condition. This sample size affords adequate (94%) power to detect small effect sizes (*d* = 0.35 for α = 0.025 after Bonferroni correction for 2 regions) for the effects of reward learning signal on brain responses. In addition, we will perform a sensitivity analysis in the inert pill + sTBS condition (*N* = 40) to ensure that results in the larger sample are not confounded by the opioid manipulation. This sample affords 99% power to detect large effects for the effects of reward learning signal on brain responses, like those observed in our preliminary data (Cohen’s *d* = 0.8 for α = 0.025 after Bonferroni correction for 2 regions). If neural learning signals are detected, the sample size of 120 subjects also affords adequate (90%) power to detect large effect sizes (Cohen’s *f* = 0.7, for α = 0.025) for the effects of neural learning signal on mood responses. This power was estimated based on one-way repeated-measures ANOVA; we expect greater power using LME models.

#### AIM 2: To determine a causal role for vmPFC modulated expected values in antidepressant placebo effects

**Figure 4.**
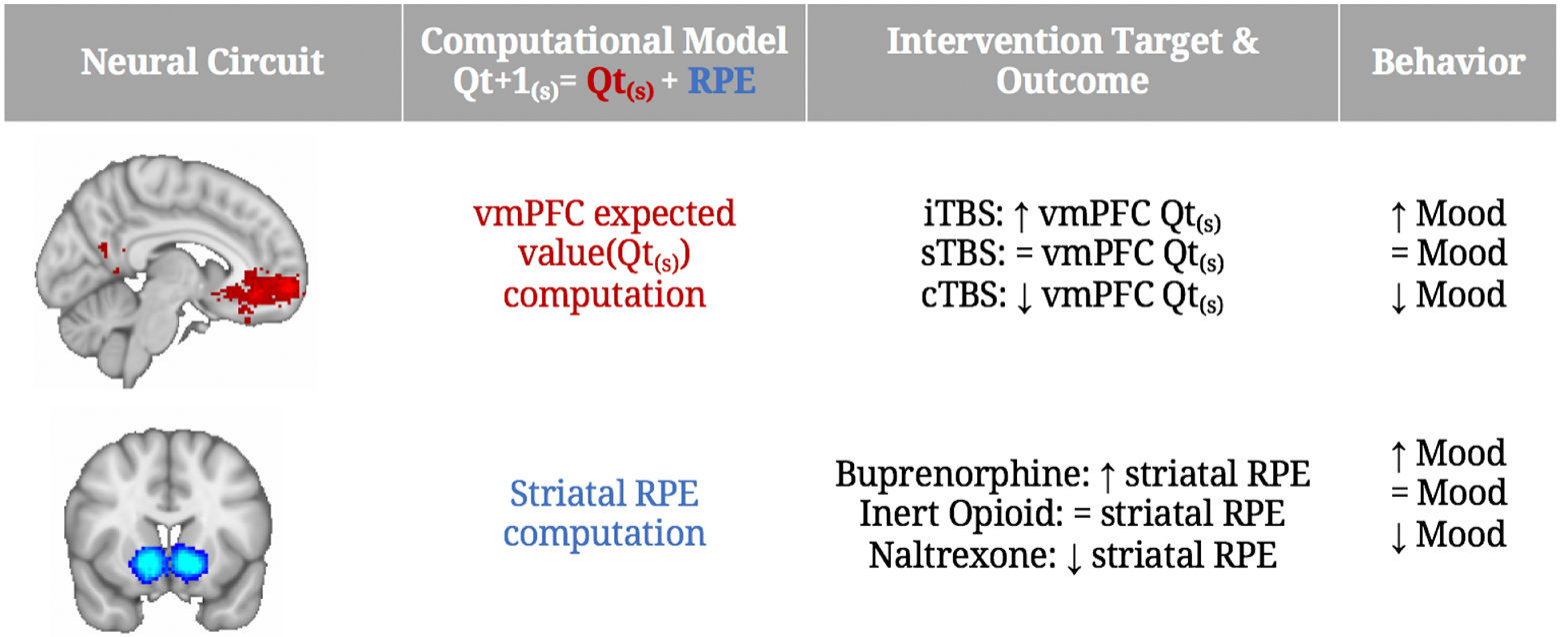
Summary of Aims & Outcomes. Images on the left are the results of automated meta-analysis from neurosynth.org using the terms vmPFC and ventral striatum. Abbreviations: Qt (expected value); vmPFC (ventromedial prefrontal cortex); RPE (reward prediction error); i/c/sTBS (intermittent, continuous, sham Theta Burst Stimulation).

The goal of Aim 2 is to modulate vmPFC expected value computation using TBS. We will examine the effects of vmPFC potentiation (iTBS) and depotentiation (cTBS), compared to no potentiation (sTBS) on expected values, vmPFC-VS neural responses and mood ratings during the Antidepressant Placebo fMRI task. Hypothesis and expected results: Compared to sTBS (*N* = 120), H2a: vmPFC iTBS (potentiation, *N* = 120) will increase the expected value representation in the vmPFC-VS circuit, enhancing mood improvement, whereas H2b: cTBS (de-potentiation, *N* = 120), will induce opposite effects (Figure 4).

##### Statistical analysis:

TBS effects on expected value computations: Using the level 1 models described for aim 1, we will conduct a paired t-test of the expected value effect difference between the iTBS or cTBS and the sTBS condition using FEAT and randomize, with threshold-free cluster enhancement type I error control [76]. Significant average BOLD responses within the vmPFC-VS circuit will be extracted for statistical analysis. If voxel-wise analyses do not yield statistical significance, we will use an ROI approach, focusing on the vmPFC and VS (H2a & b). TBS modulation and mood prediction: To assess whether the effects of TBS interact with the task conditions and vmPFC-VS BOLD responses to enhance mood ratings, we will conduct a logistic mixed-effects regression analysis using the same methods described for Aim 1. In this case, fixed effects for the prediction of mood ratings will include the task conditions, vmPFC-VS BOLD responses, the TBS condition (iTBS/cTBS vs sTBS) and their interactions (H2a & b). Alternative outcomes: If cTBS acutely modulates the vmPFC as in a previous study [60] while iTBS fails to do so, Aim 2 can be accomplished comparing cTBS to iTBS, or examining linear effects (iTBS > sTBS > cTBS). If either TBS arm produces no reliable modulation of the vmPFC-VS pathway during the Antidepressant Placebo Task in the first *n* = 5, we will consider stimulating the vlPFC.

##### Power analysis [80].

Meta-analytic effect sizes for acute effects of iTBS and cTBS on motor evoked potentials suggest very large effects on the brain within the 60 min training window we propose (*d* = 1.5–2.2 for sham vs active TBS)[64]. In a recent study, vmPFC cTBS vs sTBS had moderate effects sizes in frontostriatal circuits, with the largest attenuation effect in the left caudate (*d* = −0.5) and left insula (*d* = −0.7)[60] using an ROI analysis. Our sample size of N=120 per group (iTBS/cTBS vs sTBS) affords 99% power to detect moderate effects sized (*d* = 0.5 for α = 0.025 after Bonferroni correction for 2 regions) for the effect of TBS on brain signal; and 90% power to detect large effect sizes (*f* = 0.6, for α = 0.025 after Bonferroni correction for 2 regions) for the effects of neural learning signal on mood responses. This power was estimated based on two-way repeated-measures ANOVA; however, we expect to have greater power using LME models.

#### AIM 3: To establish a causal role for μ-opioid modulated RPEs in antidepressant placebo effects

The goal of Aim 3 is to modulate striatal RPE signal using μ-opioid pharmacological approaches. We will examine the effects of the partial μ-opioid agonist buprenorphine and the μ-opioid antagonist naltrexone on striatal RPE neural responses. Hypothesis and expected results: Compared to the inert pill condition (*N* = 40 × 3 session/subject), H3a: the partial μ-opioid agonist buprenorphine (*N* = 40 × 3) will be associated with increased striatal RPEs, enhancing mood improvement, whereas H3b: the μ-opioid antagonist naltrexone (*N* = 40 × 3) will induce the opposite effects (Figure 4).

##### Statistical analysis:

μ-Opioid effects on RPE computations: Using the level 1 models described for aim 1, we will conduct a paired *t*-test of the RPE regressor between the opioid (buprenorphine or naltrexone) and the inert pill condition, using FEAT and randomize [75] with stringent type I error control [76]. Significant average striatal BOLD responses will be extracted for the prediction of mood responses. If voxel-wise analyses do not yield statistical significance, we will use an ROI approach, focusing on the VS (H3a & b) as described for Aim 2, but in this case, we will replace the TBS condition with the opioid condition (H2a & b). Alternative outcomes: If naltrexone acutely modulates RPE as in our previous study while buprenorphine fails to do so, we will increase our power by examining linear effects (buprenorphine > inert pill > naltrexone).

##### Power analysis [80].

Our sample size of *N* = 40 per group for the buprenorphine/naltrexone vs. inter pill comparison affords adequate (90%) power to detect large effect sizes (*d* = 0.7 for α = 0.05) for the effects of reward learning signal on brain signal. Given the multiple scanning sessions per subject (*3), we still afford 90% power to detect moderate effect sizes (*d* = 0.5 for α = 0.05) when all sessions are considered, after controlling for the TBS condition (*N* = 120 per group). The sample size of 40 subjects per group affords adequate (90%) power to detect large effect sizes (*f* = 0.5 for α = 0.05) for the effects of striatal learning signal on mood responses. Power was estimated based on repeated-measures ANOVA, but we expect greater power using LME models.

Sensitivity/exploratory analyses will be applied to all hypotheses to explore main and moderating effects of key biological variables: age, baseline clinical variables, and demographic variables (e.g., ethnicity), as well as potential variables of interest, such as sex [82] (~60% female expected), personality traits [83], trauma history, comorbidity and prior history of antidepressants. Additional exploratory neuroimaging analyses will test TBS by opioid interactions using mixed-effects designs in FSL using FLAME1+2 [84] (for each cell *N* = 40, see Power Analysis for AIM3 if between-subject comparison; greater power is expected in a within-subject comparison).

### Future Directions

The resulting mechanistic study will inform the development of novel treatments for depression, including weekly/daily vmPFC brain stimulation or synergistic treatment combinations (e.g., weekly/daily iTBS ± buprenorphine). Future research will also examine the potential for weekly/daily cTBS (±naltrexone) to reduce placebo effects in clinical trials. We will also examine individual differences (e.g., personality traits, trauma history, comorbidity, history of previous treatment) in RL models of antidepressant placebo effects. Finally, future research will investigate the transdiagnostic validity of RL theories of placebo effects in other clinical conditions (e.g., anxiety disorders, schizophrenia, substance use disorders).

## ABBREVIATIONS

BOLD: blood-oxygen-level dependent
i/c/sTBS: intermittent, continuous, sham Theta Burst Stimulation
I.M.: Intramuscular
I.V.: Intravenous
MDD: Major Depressive Disorder
RCT: Randomized Clinical Trials
RL: Reinforcement Learning
RMT: Resting Motor Threshold
RPE: Reward Prediction Error
TMS: Transcranial Magnetic Stimulation
VS: Ventral Striatum
vmPFC: ventromedial prefrontal cortex
